# Lysosomes and Cancer Progression: A Malignant Liaison

**DOI:** 10.3389/fcell.2021.642494

**Published:** 2021-02-26

**Authors:** Eda R. Machado, Ida Annunziata, Diantha van de Vlekkert, Gerard C. Grosveld, Alessandra d’Azzo

**Affiliations:** ^1^Department of Genetics, St. Jude Children’s Research Hospital, Memphis, TN, United States; ^2^Department of Anatomy and Neurobiology, College of Graduate Health Sciences, University of Tennessee Health Science Center, Memphis, TN, United States

**Keywords:** lysosome movement, lysosome positioning, lysosomal exocytosis, lysosomal membrane contact sites, cancer progression

## Abstract

During primary tumorigenesis isolated cancer cells may undergo genetic or epigenetic changes that render them responsive to additional intrinsic or extrinsic cues, so that they enter a transitional state and eventually acquire an aggressive, metastatic phenotype. Among these changes is the alteration of the cell metabolic/catabolic machinery that creates the most permissive conditions for invasion, dissemination, and survival. The lysosomal system has emerged as a crucial player in this malignant transformation, making this system a potential therapeutic target in cancer. By virtue of their ubiquitous distribution in mammalian cells, their multifaced activities that control catabolic and anabolic processes, and their interplay with other organelles and the plasma membrane (PM), lysosomes function as platforms for inter- and intracellular communication. This is due to their capacity to adapt and sense nutrient availability, to spatially segregate specific functions depending on their position, to fuse with other compartments and with the PM, and to engage in membrane contact sites (MCS) with other organelles. Here we review the latest advances in our understanding of the role of the lysosomal system in cancer progression. We focus on how changes in lysosomal nutrient sensing, as well as lysosomal positioning, exocytosis, and fusion perturb the communication between tumor cells themselves and between tumor cells and their microenvironment. Finally, we describe the potential impact of MCS between lysosomes and other organelles in propelling cancer growth and spread.

## Introduction

Lysosomes comprise a highly heterogeneous group of acidic organelles, enclosed by a single unit membrane, whose function is defined by their name derived from the Greek word for “digestive body”. They vary in number, shape, size and content and their biogenesis is transcriptionally and epigenetically regulated (Saftig and Puertollano, 2020). Lysosomes mature from endosomes, move along the cell’s cytoskeleton, undergo fusion and fission events and transient kiss-and-run contacts with other membranes (Luzio et al., 2007). Although catabolism of macromolecules and recycling of their breakdown products remain the primary task of lysosomes, many additional cellular processes have been assigned to this organellar system, which are nonetheless mostly driven by its digestive capacity. These include signaling, metabolic activity, lipid homeostasis, PM repair and remodeling of the extracellular matrix (ECM) (Pu et al., 2016; Davidson and Vander Heiden, 2017; Platt et al., 2018; Saffi and Botelho, 2019; Saftig and Puertollano, 2020). The way lysosomes orchestrate these functions is determined by their soluble/membrane constituents, and their intracellular localization. Eukaryotic cells contain hundreds of these organelles, but sub-pools of lysosomes with specific tasks may locate preferentially at the cell periphery or the perinuclear region. This subcellular distribution is a regulated process that depends on cell polarity, variation in cytosolic or lysosomal pH, type of membrane proteins that attach the organelles to the cytoskeleton, as well as specific physiological/pathological or environmental stimuli, and the differentiation state of the cell or tissue (Pu et al., 2016).

**FIGURE 1:**
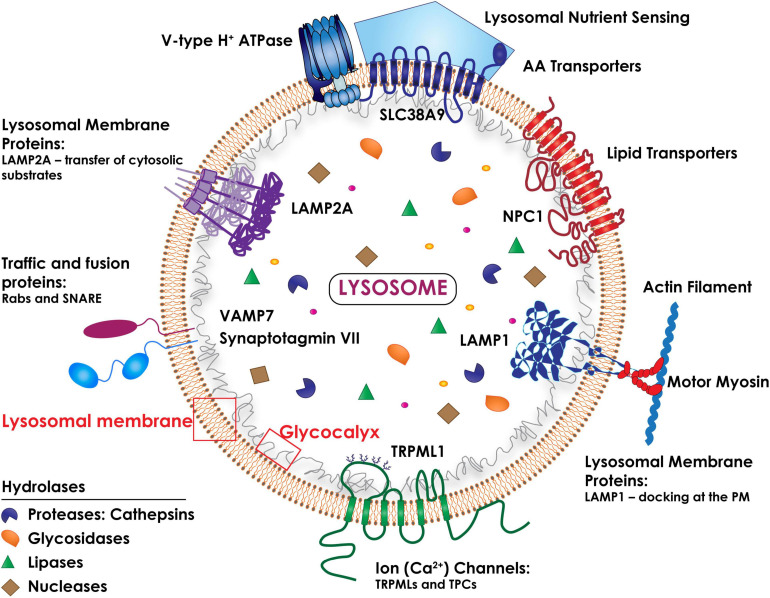
Schematic drawing of the topology of the lysosome. The lysosomal membrane comprises several integral membrane proteins (i.e., LAMP1 and LAMP2A), ion (Ca^2+^) channels (i.e., TRPMLs and TPCs), traffic and fusion proteins (i.e., Rabs and the SNARE subunits, Synaptotagmin VII and VAMP7), lipid and amino acid transporters (NPC1 and SLC38A9). The lysosomal luminal domains of the LAMPs are heavily glycosylated/sialylated, forming a protective glycocalyx that ensures the integrity of the lysosomal membrane. The multimeric vacuolar H^+^ ATPase pump is essential for maintaining the acidic pH of the lysosomal lumen needed for the activity of all lysosomal hydrolases. A multiprotein complex (lysosomal nutrient sensing) assembled at the lysosomal membrane regulates mTORC1 activity.

Macromolecular substrates reach the lysosomes via the biosynthetic, endocytic, autophagic and phagocytic routes. Their catabolism is controlled by a battery of more than 60 intraluminal hydrolases that function at a strictly acidic pH range (4.5–5.0) (Platt et al., 2018; Figure 1). The lysosome single-unit membrane embeds more than 200 integral membrane proteins, which include a vacuolar H^+^ ATPase pump (v-ATPase) that maintains the acidity of the organelles, as well as ion channels, lipid transporters, receptors, solute carriers and signaling complexes (Mindell, 2012). In addition, numerous lysosomal membrane proteins (LAMPs) are heavily glycosylated/sialylated and topologically oriented so that their glycan arborization faces the lumen, forming a protective glycocalyx that ensures integrity of the lysosomal membrane against the harsh hydrolytic environment (Saftig and Klumperman, 2009; Platt et al., 2018; Figure 1).

Unsurprisingly, genetic, epigenetic and posttranslational alterations that influence any of these interconnected lysosomal activities result in loss of cell, tissue and organism homeostasis and can cause disease. Prototypical examples of disorders associated with lysosomal dysfunction are the lysosomal storage diseases (LSDs), a large group of monogenic, mostly pediatric conditions characterized by complex multisystem pathology and neurodegeneration (Platt et al., 2018). However, it is now widely accepted that the lysosomal system is directly implicated also in common disorders prevalent in the adult population, such as neurodegenerative diseases and cancer.

During malignant transformation, cancer cells evolve and adapt their lysosomal system and its physiological processes to their advantage, in order to sustain their intrinsic anabolic and catabolic needs. Also, fundamental for cancer progression is the capacity of cancer cells to modify their microenvironment by hijacking the process of lysosomal exocytosis. By fusing with the PM, lysosomes expel soluble and particulate contents extracellularly and, in turn, alter the composition of the PMs, acidify the tumor microenvironment and degrade the ECM. These combined events create the most favorable conditions for cancer cell migration, invasion and metastatic spread. These aspects of cancer progression directly implicating the lysosomal system will be the focus of this review.

## Transcription Regulation of Lysosomal Biogenesis

**FIGURE 2:**
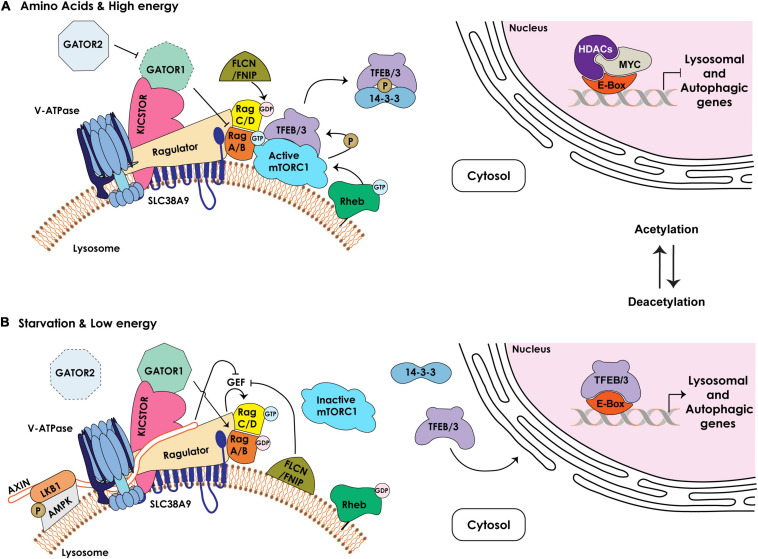
Schematic representation of the components regulating the lysosomal nutrient sensing machinery upstream of mTORC1/TFEB/TFE3. **(A)** Under nutrient rich and high energy conditions, the Ragulator/LAMTOR complex bound to the amino acid transporter SLC38A9 at the lysosomal membrane together with the v-ATPase serve as scaffold for the Rag GTPases, RagA/B and RagC/D, which cycle between an active (RagA/B^*GTP*^ – RagC/D^*GDP*^) or inactive (RagA/B^*GDP*^ – RagC/D^*GTP*^) state. The GAP activity of the GATOR1 complex, tethered to the lysosomal membrane by KICSTOR, is inhibited by the GATOR2 complex, enabling RagA/B^*GTP*^–mediated recruitment of mTORC1 to the lysosomal membrane. The FLCN/FNIP GAP activity towards RagC/D facilitates mTORC1 recruitment. The lysosome-anchored Rheb GTPase in its GTP-bound state mediates the activation of mTORC1 kinase that phosphorylates TFEB/TFE3 (TFEB/3), promoting their cytosolic retention and sequestration by the 14-3-3 proteins. In the nucleus, MYC/HDAC occupy the E-box/CLEAR binding site in the proximal promoters of lysosomal and autophagic genes, inhibiting their expression. **(B)** Under low nutrient and energy conditions the Rag GTPases are in an inactive state (RagA/B^*GDP*^ – RagC/D^*GTP*^). Active GATOR1 converts RagA/B to their GDP-bound state, which inhibits mTORC1 recruitment to the lysosomal membrane. In addition, the Ragulator /v-ATPase become accessible to AMPK/LKB1/AXIN complex at the lysosomal membrane. AXIN inhibits the GEF activity of the Ragulator promoting mTORC1 dissociation. FLCN/FNIP complex, bound to the lysosomal membrane, inhibits the GEF activity of the Ragulator and switches the Rag GTPases to an inactive state, leading to dissociation of mTORC1 from the lysosomal surface and its inhibition. Dephosphorylated TFEB/3 is released from 14-3-3 and translocates to the nucleus. Inhibition of HDAC and acetylation of histones reduce c-MYC levels and allow for the binding of TFEB/3 to the E-boxes/CLEAR sequence, resulting in the transcriptional activation of lysosomal and autophagic genes.

Lysosomal biogenesis is controlled by coordinated transcription and epigenetic programs, which play a critical role in cancer metabolism and progression (Miranda-Goncalves et al., 2018; Perera et al., 2019; Figure 2). Transcription of lysosomal and autophagic genes is regulated by the activity of the MiT/TFE (microphthalmia-transcription factor E) basic helix-loop-helix (bHLH) leucine zipper family of transcription factors, comprising MITF, TFEB, TFE3 and TFEC (Saftig and Puertollano, 2020). MiT/TFE family members are differentially expressed in different cell types and operate both as homodimers and heterodimers (Raben and Puertollano, 2016; Yang et al., 2018). All 4 recognize a unique E-box (enhancer box) DNA motif (also named CLEAR for coordinated lysosomal expression and regulation) within the proximal promoters of lysosomal and autophagic genes, thereby activating their transcription (Sardiello et al., 2009; Settembre et al., 2011; Saftig and Puertollano, 2020; Figure 2). However, the precise regulation of lysosomal biogenesis and autophagy likely requires a much more sophisticated interplay between MiT/TFE and transcriptional repressors than currently known. Those identified to date are two master regulators of autophagy, proliferation and metabolism, ZKSCAN3 (zinc finger protein with KRAB and SCAN domains 3) and c-MYC (Chauhan et al., 2013; Annunziata et al., 2019). Adding to the complexity of this regulatory network is the fact that MiT/TFE transcription factors themselves, as well as lysosomal and autophagic genes, are epigenetically controlled by histone deacetylases (HDAC). Specifically, it was demonstrated that HDAC2 in association with c-MYC transcriptionally competes with the MiT/TFE members, TFEB and TFE3, by binding to the same E-box/CLEAR sequence in the promoters of lysosomal and autophagic genes, which represses their transcription (Annunziata et al., 2019; Figure 2). Pharmacologic or genetic inhibition of HDAC abolishes binding of c-MYC to the promoter of lysosomal and autophagic genes, allowing MiT/TFE members to occupy the same binding site and activate their transcription. The net consequence of this regulatory rheostat is the rapid and dynamic modulation of the lysosomal system in response to a myriad of extracellular and intracellular signals, including starvation, inflammation, ER and oxidative stress, and mitochondrial damage. This is particularly relevant in the context of cancer, because chronic activation of any of the above-mentioned stressors that perturb lysosomal function can fuel cancer progression (Davidson and Vander Heiden, 2017).

## Lysosomal Adaptation

In response to specific intra- or extracellular cues, TFEB/TFE3, the most studied members of the MiT/TFE family, shuttle between the cytosol, the lysosomal membrane and the nucleus through cycles of phosphorylation/dephosphorylation of specific serine residues (Puertollano et al., 2018; Ballabio and Bonifacino, 2020; Saftig and Puertollano, 2020). One of the best characterized kinases that phosphorylates TFEB/TFE3 is the lysosome-associated Ser/Thr kinase mTOR (mechanistic target of rapamycin), as part of the mTORC1 complex (Efeyan et al., 2012; Puertollano, 2014; Rabanal-Ruiz and Korolchuk, 2018). The function of mTORC1 is intimately connected to the lysosome (Figure 2). For its activation mTORC1 needs to be recruited to the membrane of a pool of lysosomes, localized in the vicinity of the PM, through interaction with Rag (Ras-related guanosine triphosphatase-binding protein) GTPases and consequent association with Rheb (Ras homolog enriched in brain) GTPases. Rag GTPases are themselves regulated by the so called Ragulator (also known as LAMTOR), a multiprotein complex also localized to the lysosomal membrane. The Rag GTPases consist of two obligate heterodimers, RagA or RagB bound to either RagC or RagD. These heterodimers cycle between their GTP/GDP-bound state that is dictated by nutrient availability. In response to specific amino acids or in nutrient rich conditions, the Ragulator transfers GTP onto RagA/RagB (RagA/B^*GTP*^ - RagC/D^*GDP*^), which in this conformation can bind to and recruit mTORC1 to the lysosomal membrane (Bar-Peled et al., 2013; Figure 2). Interestingly, the activity of the Ragulator towards RagA/B depends on its interaction with the v-ATPase proton pump, which connects mTORC1 activity to changes in the lysosomal pH (Zoncu et al., 2011).

Other activators or repressors have been identified that modulate the activity of RagA/B-RagC/D and, in turn, mTORC1 in response to amino acid or energy levels. These include the octomeric GATOR (GTPase activating proteins [GAP] toward Rags) complex, composed of two subcomplexes GATOR1 and 2, which regulate the pathway that signals amino acid availability to mTORC1. The GATOR1 subcomplex exerts GAP activity towards RagA/B, promoting their inhibition, and its loss of function renders mTORC1 signaling insensitive to amino acid starvation; instead, the GATOR2 subcomplex activates Rags by inhibiting GATOR1 (Bar-Peled et al., 2013; Figure 2). Recruitment of GATOR1 to the lysosomal surface occurs in an amino acid independent manner via KICSTOR, a protein complex that also localizes to the lysosomal membrane and is necessary for the interaction of GATOR1 with the Rag GTPases (Wolfson et al., 2017). Together with the GATOR complexes, FLCN (Folliculin) in complex with FNIP1/2 (FLCN-interacting proteins 1 and 2) also functions as a GAP for RagC/D thereby mediating mTORC1 activation. Under amino acid deprivation, FLCN/FNIP1/2 interact with GDP-bound RagA, enabling mTORC1 dissociation from the lysosomal membrane (Tsun et al., 2013; Figure 2). Lastly, another key lysosomal membrane-resident protein, the SLC38A9 (solute carrier family 38 member 9), functions as a positive regulator of mTORC1 signaling by interacting with the Rag GTPases and the Ragulator complex (Wang et al., 2015). SLC38A9 acts as a lysosomal arginine sensor that, upon activation by arginine binding, transports essential amino acids (i.e., leucine, tyrosine and phenylalanine), derived from lysosomal catabolism, from the lysosomal lumen to the cytosol in an arginine-concentration dependent manner (Wang et al., 2015). Given that arginine facilitates the interaction of SLC38A9 with the Ragulator and Rag GTPases, the arginine concentration directly modulates mTORC1 activity. Recently, it was demonstrated that this solute carrier also senses cholesterol levels and binds cholesterol at specific MCS formed between lysosomes and the ER (see below). In this capacity SLC38A9 activates mTORC1 independently from arginine sensing (Tsun et al., 2013; Lim et al., 2019).

As mentioned earlier, activation of mTORC1 at the lysosomal membrane leads to phosphorylation of TFEB and TFE3, which promotes their binding to 14-3-3 proteins and retention in the cytosol (Puertollano, 2014; Puertollano et al., 2018; Figure 2). In contrast, upon starvation (specifically glucose deprivation) or under low energy conditions (increase in AMP levels), lysosomes localized to the perinuclear region recruit and activate a portion of cytosolic AMPK (5’ AMP-activated protein kinase), which simultaneously inhibits mTORC1 activity and promotes TFEB/TFE3 nuclear translocation (Zhang et al., 2014). Low energy conditions stimulate AMPK recruitment to the lysosomal membrane by binding to LKB1 (Liver Kinase B 1), which together with AXIN forms the large v-ATPase-Ragulator-AXIN/LKB1-AMPK complex. The latter association inhibits the activity of the Rag GTPases, leading to dissociation of mTORC1 from the lysosomal surface, thereby extinguishing its kinase activity (Zhang et al., 2014). Interestingly, this lysosomal sub-pool of AMPK is activated by low glucose more potently than by low AMP levels, connecting also this kinase to nutrient availability.

TFEB/TFE3’s nuclear versus cytosolic localization is also regulated by other kinases and phosphatases. These include the kinases AKT and GSK3 (Ploper et al., 2015; Palmieri et al., 2017), and the Ca^2+^ binding phosphatase calcineurin (Medina et al., 2011), see for review (Puertollano et al., 2018).

## Lysosomal Biogenesis, Adaptation and Regulation in Cancer

Given the complexity of these regulatory nodes, intimately dependent on and modulating the lysosomal system, it is not surprising that expression of the components of these pathways is reprogrammed during cancer progression. For example, the c-MYC/HDAC2-MiT/TFE transcriptional rheostat promotes the progression of colon adenocarcinoma, medulloblastoma and rhabdomyosarcoma to an aggressive, higher grade state (Annunziata et al., 2019). In these tumors, cancer cells expressing high levels of c-MYC and HDAC2 in the nucleus force relocation of TFEB/TFE3 to the cytoplasm, which inhibits lysosomal biogenesis and autophagy (Annunziata et al., 2019). In contrast, in pancreatic ductal adenocarcinoma cells, inactivation of mTORC1 and consequent translocation of MiT/TFE to the nucleus increases autophagy and lysosomal catabolism, which maintains a stable pool of amino acids essential for cell growth (Perera et al., 2015). In melanoma cells, nuclear accumulation and stabilization of MITF also results in endo-lysosomal biogenesis and increases the number of late endosomes/multivesicular bodies (MVBs) without induction of lysosomal proteolysis (Ploper et al., 2015). This increased endosome/MVB biogenesis was shown to be associated with enhanced WNT signaling due to sequestration of GSK3, ultimately contributing to melanoma proliferation (Ploper and De Robertis, 2015; Ploper et al., 2015). Similarly, kidney-specific overexpression of TFEB in transgenic mice leads to a highly cystic phenotype that progresses into papillary renal carcinoma with liver metastasis downstream of the WNT/β-catenin signaling pathway (Calcagni et al., 2016). In this model, activation of β-catenin induces strong expression of target genes, including c-MYC. Considering that these authors did not observe significant changes in the expression levels of lysosomal and autophagic genes, it is tempting to speculate that induction of c-MYC in this transgenic model had a more potent effect on cancer progression than TFEB overexpression, leading to inhibition of lysosomal biogenesis and autophagy.

Chromosome translocations involving the MiT/TFE members can generate gene fusions that have been shown to occur in several cancer types, including melanoma, clear cell sarcoma of the tendon sheath, perivascular epithelioid cell tumor, alveolar soft part sarcoma of the soft tissue, non-small cell lung cancer and renal cell carcinoma. In these tumors, increased expression of the encoded fusion protein correlates with poor outcome and metastatic disease (Kauffman et al., 2014; Argani, 2015; Durinck et al., 2015; Giatromanolaki et al., 2015; Saleeb et al., 2017). Since gene fusions involving MITF, TFEB or TFE3 preserve the open reading frame of these transcription factors and retain the DNA-binding domain (Kuiper et al., 2003), it is plausible that at least some steps in the malignant transformation associated with MiT/TFE gene fusions may also depend on the activation of endo-lysosomal biogenesis.

Last but not least, activation and deregulation of mTORC1 affecting autophagy and lysosomal biogenesis have been implicated in malignant transformation and in sustaining cancer growth, but this subject has been extensively discussed in several reviews (McCarty, 2011; Efeyan et al., 2012; Kimmelman and White, 2017; Saxton and Sabatini, 2017; Amaravadi et al., 2019; Zou et al., 2020).

## Lysosomal Positioning

**FIGURE 3:**
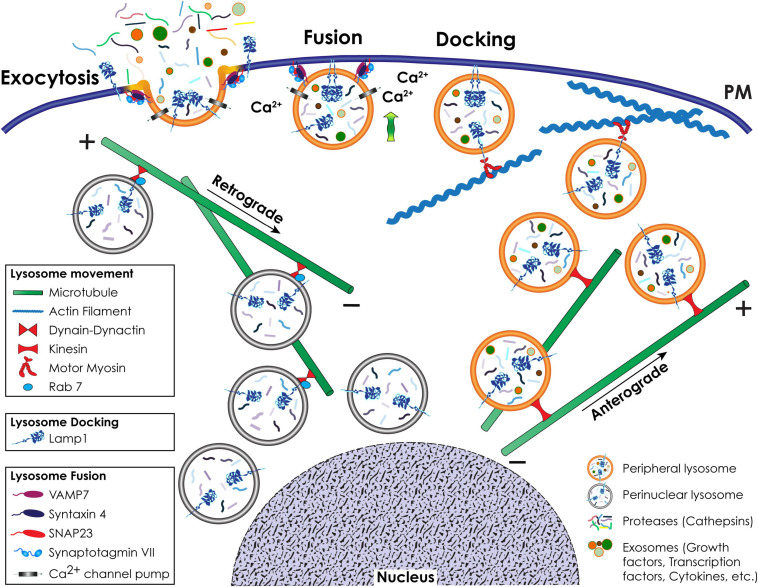
Schematic representation of lysosomal positioning and lysosomal exocytosis. Retrograde movement of a pool of lysosomes to the perinuclear region occurs toward the minus-end of microtubules and is mediated by Rab7 and the dynein-dynactin complex. Anterograde movement of lysosomes to the cell periphery occurs toward the plus-end of microtubules and is mediated by kinesin motors. Close to the cell surface, lysosomes that engage in lysosomal exocytosis move along actin filaments via interaction with a motor myosin. During lysosomal exocytosis, the docking of lysosomes at the PM is mediated by LAMP1. The fusion of the lysosomal membrane with the PM depends on the Ca^2+^ sensing activity of SytVII and is mediated by v-SNARE and t-SNARE complexes.

Lysosomal movement is a regulated process that depends on a complex network of microtubules, actin filaments and motor proteins in the cytosol of cells and membrane proteins in the lysosome (Cabukusta and Neefjes, 2018; Figure 3). This dynamic interplay is particularly important in a cancer setting where transforming cells change morphology, lose their polarity and rewire their metabolic program (Henne, 2017). Unlike in polarized cells, such as neurons and epithelial cells, in non-polarized cancer cells, lysosomes move bidirectionally along the microtubules’ minus-end near the perinuclear MTOC (microtubule organizing center), and the plus-end at the cell periphery (Pu et al., 2016; Cabukusta and Neefjes, 2018). This long-range transport is propelled by microtubule motors, such as kinesins and dynein, while myosin motors drive short-range transport, close to the PM along actin filaments (Bonifacino and Neefjes, 2017).

The retrograde (centripetal) movement of lysosomes from the plus-end of microtubules at the periphery of cells to the MTOC is dependent on the interaction between two multisubunit complexes, dynein and dynactin (Hirokawa et al., 2009; Urnavicius et al., 2015; Li X. et al., 2016; Bonifacino and Neefjes, 2017; Figure 3). Coupling of the dynein-dynactin mega complex to lysosomes is mostly dependent on the small GTPase Rab7 and its effector proteins, including RILP (Rab7-interacting lysosomal protein) and the cholesterol sensor ORPL1 (OSBP [oxysterol binding protein]-related protein 1). Besides Rab7, other effectors of the lysosome-dynein-dynactin coupling are ALG-2 (apoptosis-linked gene 2), TRPML1 (transient receptor potential mucolipin 1), LAMP1, LAMP2, TMEM106B (transmembrane protein 106B), and TMEM55B (Bonifacino and Neefjes, 2017; Ballabio and Bonifacino, 2020). These effectors mediate the coupling process under specific stress conditions. The opposite, anterograde (centrifugal) movement of lysosomes from the perinuclear region to the cell periphery is mediated by kinesin motors (Bonifacino and Neefjes, 2017; Figure 3). There are 45 mammalian kinesin genes organized in 15 superfamilies (Hirokawa et al., 2009), which reflects their cell and cargo specificity. For the efficient transport of cargo bound to their tail domain, kinesins depend on ATP hydrolysis through their globular motor domain attached to microtubules (Pu et al., 2016). Lysosomes interact with different kinesins, a process that possibly depends on cell type specific expression, different lysosomal functions and specific posttranslational modifications of components of the microtubule tracks. However, the mechanism(s) dictating the selectivity of these interactions has not been fully elucidated (Pu et al., 2016). Kinesin (KIF)1 is the best characterized kinesin involved in lysosomal movement. KIF1 forms a heterotetramer consisting of two heavy chains and two light chains that recognizes lysosome interacting complexes (Bonifacino and Neefjes, 2017). One of these complexes is composed of the multisubunit BORC (BLOC-1-related complex) (Pu et al., 2015). At the lysosomal membrane, BORC recruits and activates the small Arf-like GTPase, Arl8, which, by binding to SKIP (SKI-interacting protein), allows for kinesin-mediated lysosomal movement upon ATP hydrolysis (Rosa-Ferreira and Munro, 2011; Pu et al., 2015).

Mutations or knockdown of any of the components of this large lysosome-kinesin multiprotein complex inhibits anterograde movement and accumulates lysosomes at the MTOC (Bonifacino and Neefjes, 2017). In contrast, overexpression of proteins within this complex leads to lysosomal accumulation at the cell periphery (Bonifacino and Neefjes, 2017). An interesting finding, which connects nutrient sensing to lysosomal positioning, was that BORC also interacts with the Ragulator, which negatively regulates Arl8b-dependent lysosome positioning and movement (Filipek et al., 2017; Pu et al., 2017). These authors further demonstrated that silencing proteins of the Ragulator complex triggers peripheral localization of lysosomes. Finally, a dense network of cortical actin fibers serves as tracks for myosin motor proteins to drive lysosome movement close to the cell periphery. Small Rab GTPases regulate the tethering of more than 30 differentially expressed myosins to the actin filaments (Pu et al., 2016; Cabukusta and Neefjes, 2018). Rab proteins on the lysosomal membrane bind to their synaptotagmin-like effector proteins (Slp) and recruit myosin motors attached to the actin filaments in order to transport lysosomes to and from the PM (Cabukusta and Neefjes, 2018).

## Lysosomal Exocytosis

Those lysosomes that are juxtaposed to the PM may be already poised to undergo fusion with the PM and exocytose their content extracellularly in the process of lysosomal exocytosis. This process was initially reported in 1968 to describe the release of acid hydrolases from osteoclasts during bone resorption (Vaes, 1968). Thereafter, it was recognized as a physiological mechanism occurring only in specialized cells such as platelets, mast cells, neutrophils, cytotoxic T cells, melanocytes and macrophages (Griffiths et al., 2010; Samie and Xu, 2014) that contain secretory lysosomes, now referred to as LROs (lysosomal related organelles) (Platt et al., 2018). It is now widely accepted that lysosomal exocytosis is a ubiquitous and generalized process that occurs in virtually all cell types and executes essential functions, including PM repair and remodeling, ATP and H^+^ release, immune response and antigen presentation (Logan et al., 2003; Shin et al., 2012; Jung et al., 2013; Andrews and Corrotte, 2018; Silberfeld et al., 2020).

Lysosomal exocytosis is a Ca^2+^-regulated process that entails the recruitment of a selected pool of lysosomes to the cytoskeletal network for transport to and docking at the PM, followed by their fusion with the PM and the extracellular release of their luminal contents (Rodriguez et al., 1997; LaPlante et al., 2006; Yogalingam et al., 2008; Samie et al., 2013; Figure 3). One of the proteins responsible for the docking of lysosomes at the PM is LAMP1. This type 1 transmembrane protein has a large, heavily glycosylated/sialylated N-terminal luminal domain and a short, C-terminal cytosolic tail of 11 amino acids (Saftig and Klumperman, 2009; Platt et al., 2018). The latter is likely responsible for attaching lysosomes to the actin filaments via interaction with motor myosins, and for their docking at the PM (Kima et al., 2000; McNeil, 2002; Machado et al., 2015). Mutations changing the tyrosine or glycine residues in the LAMP1 cytosolic tail, or downregulation of LAMP1 expression, impair lysosomal exocytosis (Kima et al., 2000; Yogalingam et al., 2008), and redistribute lysosomes from the cell periphery to the juxtanuclear region (Yogalingam et al., 2008). How LAMP1’s cytosolic tail physically hooks lysosomes onto the cytoskeleton and/or the PM is still not fully understood. We hypothesize that either the amino acid sequence or posttranslational modification(s) of the cytosolic tail itself determines the type of protein that interacts with LAMP1, promoting the docking of lysosomes at the PM and lysosomal exocytosis. However, we cannot exclude that alterations in the glycan composition of the luminal domain of LAMP1 induces structural changes in its C-terminal tail, thereby influencing LAMP1 dynamics. In support of this scenario is the finding that hydrolytic removal of the sialic acids on LAMP1 glycans by the lysosomal sialidase NEU1 (neuraminidase 1) regulates the number of lysosomes that dock at the PM and in turn the extent of lysosomal exocytosis (Yogalingam et al., 2008). In cells from NEU1 deficient mice, a model of the LSD sialidosis, impaired processing of sialic acids on LAMP1’s luminal domain prolongs its half-life and results in an increased number of LAMP1-decorated lysosomes docked at the PM, poised to engage in lysosomal exocytosis (Yogalingam et al., 2008; d’Azzo et al., 2015).

Lysosome fusion at the PM is initiated by the ubiquitously expressed lysosomal synaptotagmin VII (SytVII), a Ca^2+^ sensor that is anchored to the lysosomal membrane by a single transmembrane domain, with the majority of the protein exposed to the cytosol (Figures 1, 3). Following a local Ca^2+^ spike, influxed from the PM or released from lysosomal stores (Rodriguez et al., 1997; Reddy et al., 2001; Jaiswal et al., 2002), SytVII begins the fusion process by undergoing a conformational change that promotes its interaction with the v-SNARE (soluble N-ethylmaleimide-sensitive factor attachment protein receptor) VAMP7 at the lysosomal membrane, and the t-SNARE-phospholipid interacting complex, syntaxin 4 and SNAP23 (synaptosomal associated protein 23) at the PM (Martinez et al., 2000; Rao et al., 2004; Arantes and Andrews, 2006; Figures 1, 3). Some of the Rab GTPases, i.e., Rab3a, together with its effector Slp4-a and MYHIIA (non-muscle myosin heavy chain IIA) have also been implicated in the docking and fusion steps of lysosomal exocytosis during the process of wound repair (Barr, 2013; Encarnacao et al., 2016). Fusion of the lysosomal membrane with the PM results in the redistribution of lysosomal membrane proteins in their original topological orientation at the PM, followed by the release of soluble lysosomal contents and exosomes extracellularly (Rodriguez et al., 1997; Reddy et al., 2001; Jaiswal et al., 2002; Yogalingam et al., 2008; van de Vlekkert et al., 2019). The appearance of lysosomal membrane proteins, and specifically LAMP1, at the PM of cells is now widely used as a readout of lysosomal exocytosis (Andrews, 2017; Figure 3).

## Lysosomal Positioning and Exocytosis in Cancer

**FIGURE 4:**
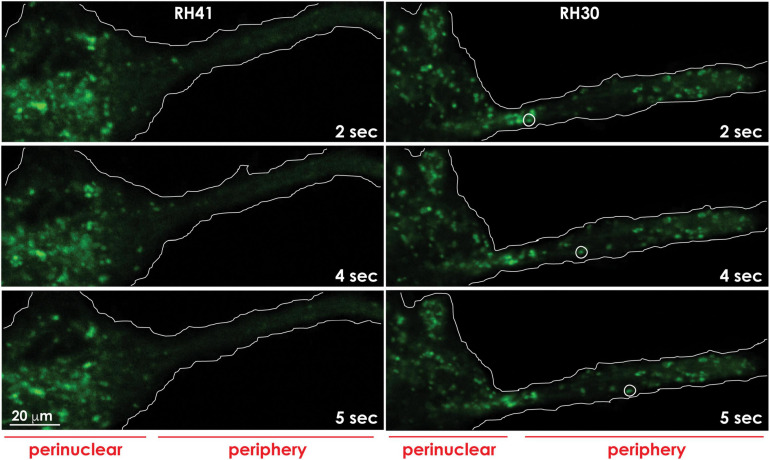
Lysosomes redistribute to the cell periphery in aggressive cancer cells. Lysosomes marked with Lysotracker green in aggressive rhabdomyosarcoma cells (RH30) move to and redistribute at the cell periphery, whereas lysosomes in less aggressive rhabdomyosarcoma cells (RH41), cluster around the perinuclear region. The movement of a lysosome (white circle) to the cell periphery in RH30 cells was recorded in a movie and snapshots were taken at the indicated timepoints. The contours of the cells are demarcated with a white line. These images are adapted from a movie published in Machado et al. (2015).

**FIGURE 5:**
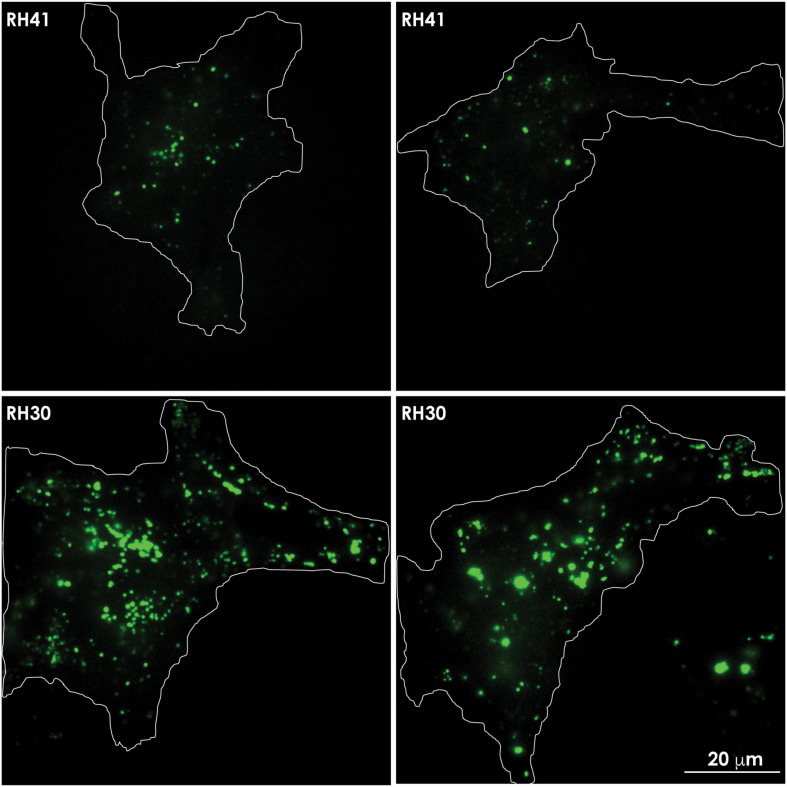
In aggressive cancer cells an increased number of lysosomes accumulates at the PM, prior to undergoing lysosomal exocytosis. Total internal reflection (TIRF) microscopy shows the presence of lysotracker green marked lysosomes in the evanescence field underneath the PM of rhabdomyosarcoma cells. Aggressive rhabdomyosarcoma cells (RH30) show an increased number of clustered lysosomes juxtaposed to the PM, compared to the number seen in less aggressive rhabdomyosarcoma cells (RH41). The contours of the cells are demarcated with a white line.

**FIGURE 6:**
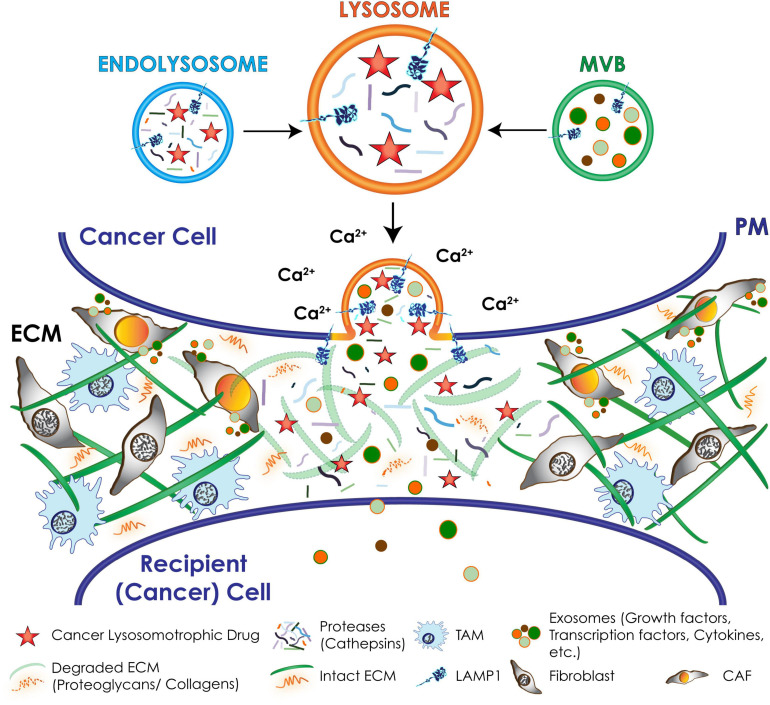
Schematic representation of downstream effects of lysosomal exocytosis in cancer cells. Upon a Ca^2+^ spike lysosomes docked at the PM undergo fusion with the PM and secrete their content extracellularly, including active hydrolases and extracellular matrix (ECM) components that remodel and degrade the ECM; chemotherapeutic drugs that are weak-bases and tend to accumulate in the acidic lysosomes. Exosomes packed with invasive signaling molecules are released via lysosomal exocytosis by cancer cells and induce the transformation of resident fibroblasts and macrophages of the tumor microenvironment into cancer associated fibroblasts (CAFs) and tumor associated macrophages (TAMs).

During cancer progression, lysosomes tend to relocate at the PM because of changes in the cytoskeletal network and/or lysosomal trafficking and exocytosis (Sameni et al., 1995; Nishimura et al., 1998; Glunde et al., 2003; Machado et al., 2015; Figures 4, 5). Cancers hijack these molecular events to become aggressive and intractable. Increasing lysosomal exocytosis empowers cancer cells in multiple ways: (1) it alters the PM makeup, thereby influencing signaling events that trigger metabolic and morphologic changes and lead to survival and migration; (2) it secretes active hydrolases and ECM components that remodel the surrounding matrix and activate stroma resident cells; (3) it enhances the release of exosomes propagating signaling molecules to neighboring cells; (4) it confers drug resistance by promoting the efflux of lysosomotropic chemotherapeutics (Figure 6 and see below). Evidence of the effects of deregulated lysosomal exocytosis on malignant transformation have been shown in human rhabdomyosarcoma cells with low NEU1 expression. In these cells, lysosomes decorated with a fully sialylated LAMP1 preferentially move to the cell periphery and dock at the PM via LAMP1-mediated interaction with the motor myosin MYH11, ready to exocytose their contents (Figures 4, 5). In addition, these rhabdomyosarcoma cells become migratory and invasive by promoting ECM degradation, and propagate oncogenic signals to neighboring cells through the release of tumor exosomes (Figure 6). They also become chemoresistant by entrapping lysosomotropic chemotherapeutics that are preferentially released by lysosomal exocytosis (Machado et al., 2015; Figure 6). Unsurprisingly, increased expression levels of LAMP1 have been correlated with tumor grade, metastatic potential and poor prognosis in many cancers, including breast and colon carcinoma, high grade glioma, and metastatic melanoma (Saitoh et al., 1992; Agarwal et al., 2015; Alessandrini et al., 2017; Wang et al., 2017; Sarafian et al., 2018). In these cases, increased LAMP1 Could also be the result of downregulation of NEU1.

Although repositioning of lysosomes at the cell periphery has not always been interpreted as a prelude to lysosomal exocytosis, we can infer that many of the changes in the cytoskeletal network and lysosomal trafficking machinery that occur during cancer progression are used by tumor cells to hijack lysosomal exocytosis. In support of this argument, numerous studies report the presence of various lysosomal membrane proteins at the PM of invasive cancer cells. For instance, one study in breast cancer reports the presence of LAMP2, a LAMP1 homologous protein, at the PM of tumor cells located at the invasive front. These authors suggest that LAMP2 redistribution at the PM is an adaptive mechanism that allows cancer cells to survive in a harsh, acidic microenvironment by forming a protective glycocalyx that circumvents acid-induced proteolysis of the PM (Damaghi et al., 2015). However, an alternative hypothesis could be that unrestrained lysosomal exocytosis is the culprit that drives both the redistribution of LAMP2 at the PM and contributes to the acidification of the tumor microenvironment. Another example of the consequences of deregulated lysosomal exocytosis is that redistribution of LAMP1 and LAMP2 at the PM of cancer cells promotes tumor invasion and metastasis via the interaction of their glycan exposed domains with galectins and selectins (Saitoh et al., 1992; Sawada et al., 1993; Dange et al., 2015; Sarafian et al., 2018).

Preferential movement of lysosomes to the periphery of cancer cells occurs as a consequence of downregulation of Rab7, which inhibits retrograde trafficking of the organelles to the perinuclear region. In several cancer types, such as prostate cancer and melanoma, redistribution of lysosomes at the PM has been shown to cause the extracellular release of lysosomal proteases, namely cathepsins, likely via lysosomal exocytosis, which remodels the ECM and facilitates tumor growth and invasion (Rozhin et al., 1994; Nishimura et al., 1998; Steffan et al., 2009; Steffan et al., 2014). In prostate cancer cells and xenografts, the combination of increased HGF (hepatocyte growth factor) and EGF (epidermal growth factor) production, combined with the acidic extracellular pH, triggers signaling events that elevate the expression of Arl8b. This causes anterograde movement of lysosomes to the cell surface and release of lysosomal proteases, leading to cancer cell invasion (Dykes et al., 2016). In line with these results is the observation that relocation of lysosomes at the PM is controlled by the relative concentration of Rab7 and Arl8b, with peripheral lysosomes containing more Arl8b and less Rab7 (Johnson et al., 2016). Therefore, it follows that, during cancer progression, changes in the expression levels of other Rab GTPases, e.g., Rab25, Rab26, Rab27, and Rab37 (Tzeng and Wang, 2016), will also stimulate lysosomal movement to the cell periphery. For example, upregulation of Rab25 promotes localization of lysosomes loaded with α5β1 integrin to the PM at the tips of pseudopodia, which facilitates migration and invasion of ovarian carcinoma cells through a fibronectin-rich ECM (Cheng et al., 2004; Caswell et al., 2007). In breast cancer cells, increased expression of Rab27b was shown to regulate growth and metastasis by promoting lysosomal secretion of HSP90a (heat-shock protein 90a) and, in turn, activation of MMP2 (matrix metalloprotease 2), which degrades the extracellular collagen and facilitates invasion (Hendrix et al., 2010; Quintero-Fabian et al., 2019). Also, alterations in microtubules’ motor proteins have been shown to mediate the movement of lysosomes to the periphery of aggressive cancer cells. Knockdown of KIF20A and KIF25, tropomyosin 2 and MYH1 in the moderately aggressive breast cancer cell line MCF7 causes increased lysosomal volume and relocation of lysosomes to the cell periphery and protrusions, rendering these cancer cells more invasive (Groth-Pedersen et al., 2012).

## Lysosomal Exocytosis and Tumor Microenvironment

During cancer progression, another potential consequence of excessive lysosomal exocytosis in selected cancer cells is the activation of stromal cells within the tumor microenvironment. Stromal cells consist of cancer associated fibroblasts (CAFs), innate/adaptive immune cells, including the tumor associated macrophages (TAMs), and vascular endothelial cells and pericytes (Quail and Joyce, 2013). By exploiting the lysosomal system, and in particular lysosomal exocytosis, stromal cells can effectively synergize with tumor cells to deposit large quantities of ECM components (e.g., collagen, laminin, fibronectin, and proteoglycans), and release proteases (e.g., MMPs and cathepsins), and oncogenic signaling molecules (e.g., cytokines and growth factors) (Figure 6). Together, these events control several aspects of cancer progression, including angiogenesis, tumor cell migration, invasion, and metastatic spread (Olson and Joyce, 2015; Brassart-Pasco et al., 2020). These pro-malignant processes, which are mostly fueled by CAFs and TAMs, transform the tumor microenvironment into a fibrotic desmoplastic state, a hallmark of aggressive and intractable cancers (Erez et al., 2010; Afik et al., 2016; Kalluri, 2016). CAFs and TAMs are capable of propagating and perpetuating oncogenic signals to neighboring and distant sites by releasing growth factors and cytokines, such as EGF and TGFβ (transforming growth factor β) (Quail and Joyce, 2013; Noy and Pollard, 2014). In a number of cancers these signaling molecules have been found to package into exosomes, which propagate these signals to neighboring cells promoting tumor growth and metastatic spread (Peinado et al., 2012; Mu et al., 2013; Hoshino et al., 2015; Kanada et al., 2016; Sung and Weaver, 2017). Canonically, exosomes compose the MVBs and are released extracellularly by fusion of the MVBs with the PM (Kalluri and LeBleu, 2020). However, in the course of malignant transformation, exocytic cancer cells as well as CAFs may expel a large quantity of exosomes via lysosomal exocytosis. It is possible that in cells with upregulated lysosomal exocytosis the increased number of lysosomes that are positioned in the proximity of the PM fuse with the MVBs at this site, immediately prior to exocytosing their contents. Indeed, this was demonstrated in aggressive rhabdomyosarcoma cells with low expression of NEU1, where increased lysosomal exocytosis led to excessive release of exosomes carrying pro-tumorigenic signals (Machado et al., 2015). Although this has been so far the only example of lysosome-mediated exocytosis of exosomes in cancer, this process has been implicated in the shedding of extracellular vesicles from melanocytes (Waster et al., 2016), adipocytes (Kim et al., 2019), and fibroblasts in other stress and disease conditions (van de Vlekkert et al., 2019). Specifically, fibroblasts deficient for NEU1 with exacerbated lysosomal exocytosis bear features of activated fibroblasts or myofibroblasts, resembling CAFs. These cells are proliferative, migratory and secrete large numbers of exosomes loaded with TGFβ and WNT/β-catenin pro-fibrotic signals, which amplify and propagate a fibrotic state (van de Vlekkert et al., 2019). By analogy, CAFs associated with different tumor types, may use excessive lysosomal exocytosis to disseminate fibrotic, desmoplastic signals that fuel cancer progression.

## Targeting Lysosomes for Cancer Therapy

The dependence of cancer cells on the lysosomal system for transitioning into a more aggressive state, makes these organelles an attractive therapeutic target. So far, lysosome-driven cytotoxicity has mostly focused on the ability of lysosomes to leak harmful hydrolases, particularly cathepsins (i.e., cathepsins B and D) into the cytosol by lysosomal membrane permeabilization (LMP) (Wang et al., 2018). LMP can be triggered by the formation of reactive oxygen species (ROS) or reactive iron (Fe^2+^), both of which result in lysosomal lipid and protein peroxidation by the formed reactive hydroxyl radicals. In addition, cleavage and disruption of lysosomal membrane proteins by cytosolic proteases also result in LMP. The type of cell death provoked by minor damage of the lysosomal membrane and small-scale leakage of cathepsins into the cytosol differs depending on the effectors: apoptosis (activation of Bax and ROS), pyroptosis (ROS) and ferroptosis (Fe^2+^ and ROS) (Wang et al., 2018). In contrast, complete rupture of the lysosomal membrane and massive discharge of lysosomal proteases into the cytosol result in cell death by necrosis (Groth-Pedersen and Jaattela, 2013). One example of a chemotherapeutic that mediates LMP-dependent death of cancer cells is the thiosemicarbazone, Dp44mT (di-2-pyridylketone 4,4-dimethyl-3-thiosemicarbazone) (Whitnall et al., 2006). This compound accumulates in lysosomes of cancer cells, where, by binding to metal ions such as iron or copper, it forms a complex that triggers ROS production and LMP-dependent cell death (Lovejoy et al., 2012; Jansson et al., 2015). In pancreatic cancer cells, Dp44mT treatment has been shown to increase the nuclear translocation of TFEB and lysosomal biogenesis, therefore augmenting the targetable surface of this drug (Krishan et al., 2016). More recently, the same authors have demonstrated that Dp44mT-dependent lysosomal membrane destabilization prevents mTORC1 assembly, decreasing cell metabolism and inhibiting growth and proliferation (Krishan et al., 2020). Thus, within the group of chemotherapeutics used for cancer treatment, several have been described that promote LMP-mediated cancer cell death (Table 1); a few of them are in clinical trials (Verbaanderd et al., 2017).

**TABLE 1:** Examples of drugs that destabilize lysosomes in cancer.

Compound	Action	Examples
Verapamil	Ca^2+^ channel blocker	• Inhibits lysosomal exocytosis independent of Pgp and promotes doxorubicin cytotoxicity in rhabdomyosarcoma cells (Machado et al., 2015). • Promotes vascularization/angiogenesis in lung and pancreatic cancer; combination therapy improves gemcitabine cytotoxicity (Wong et al., 2015). • Potentiates sorafenib cytotoxicity in hepatocellular carcinoma (HCC) cells (Colombo et al., 2014).
Mefloquine	Antimalarial drug that causes LMP; inhibits autophagy	• Provokes LMP and cathepsin mediated cell death of myeloid leukemia (AML) cells (Sukhai et al., 2013). • Disrupts lysosomes and increases ROS, inhibiting growth of chronic myeloid leukemia (CML) cells; selectively increases cytotoxicity of BCR-ABL tyrosine kinase inhibitors in CML stem/progenitor cells (Lam Yi et al., 2019). • Combined with sorafenib, reverts resistance of hepatocellular carcinoma (HCC) cells (Colombo et al., 2014). • Inhibits prostate cancer cell growth by increasing LMP and ROS mediated cell death (Yan et al., 2013). • Inhibits autophagy and caused cell death in breast cancer cell lines (Sharma et al., 2012). • Increases doxorubicin cytotoxicity in a MDR cancer cell line by inhibition of Pgp efflux (Fujita et al., 2000).
Chloroquine	Antimalarial drug that causes LMP; increases lysosomal pH; inhibits autophagy by inhibiting lysosome-autophagosome fusion	• Combination therapy improves mid-term survival for glioblastoma multiforme patients (Sotelo et al., 2006). • Renders drug-resistant breast cancer cells sensitive to cyclin-dependent kinase CDK4/6 inhibitors (Fassl et al., 2020). • Potentiates therapeutic activity in combination with other chemotherapeutics in several cancer cell types (Verbaanderd et al., 2017). • Potentiates cisplatin efficacy in lung cancer cells (Circu et al., 2017). • Causes cytotoxicity in highly metastatic bladder cancer cell lines (Morgan et al., 2018). • The chloroquine analog EAD1 causes LMP mediated apoptosis, disrupts mTORC1-lysosome interaction and blocks lung cancer cell proliferation (Sironi et al., 2019).
Hydroxychloroquine	Inhibits autophagy by impairing lysosomal fusion with the autophagosome	• Potentiates therapeutic activity when in combination with other chemotherapeutics in several cancer cell types (Verbaanderd et al., 2017).
CADs (cationic amphiphilic drugs)	Destabilize lysosomal membrane by inhibiting the function of lysosomal lipases (e.g., acid sphingomyelinase)	• Loratadine, astemizole and ebastine sensitize non-small cell lung cancer (NSCLC) cells to chemotherapy and revert MDR in breast and prostate cancer cells; associate with reduced mortality of NSCLC patients (Ellegaard et al., 2016).
Bafilomycin A1	v-ATPase inhibitor; inhibits lysosomal acidification and lysosomal fusion	• Blocks autophagic flux, inhibits growth and mediates cell death in pediatric B-cell acute lymphoblastic leukemia cells (Yuan et al., 2015). • Increases cisplatin cytotoxicity in tongue squamous cell carcinoma (TSCC) cells by inhibiting lysosomal uptake of platinum and enhancing intracellular platinum ion binding to DNA (Chu et al., 2018). • Causes cytotoxicity in highly metastatic bladder cancer cell lines (Morgan et al., 2018). • Induces LMP mediated apoptosis by alkalinization and lysosomal dysfunction in gastric cancer cell lines (Nakashima et al., 2003).
Omeprazole	v-ATPase inhibitor	• Provokes production of ROS that precedes alkalinization of lysosomal pH and LMP mediated apoptosis in leukemia cell lines (De Milito et al., 2007).
Hsp70 inhibitors	Destabilize lysosomes causing LMP; impair autophagy	• Apoptozole is cytotoxic to several cancer cell lines (Park et al., 2018). • 2-phenylethynesulfonamide (PES) treatment induces cytotoxicity in primary effusion lymphoma (PEL) cells by LMP mediated apoptosis (Granato et al., 2013).
DP44mT (di-2-pyridylketone 4,4-dimethyl-3-thiosemicarbazone)	Iron chelator that induces ROS production and LMP mediated apoptosis	• Inhibits pancreatic cell growth by decreasing mTORC1 activity (Krishan et al., 2020). • Releases doxorubicin stored in lysosomes and potentiates its effect in cervical, breast and colorectal cancer cells (Seebacher et al., 2016). • Inhibits growth of osteosarcoma, melanoma and acute leukemia cells (Whitnall et al., 2006; Noulsri et al., 2009; Li P. et al., 2016). • Potentiates the effect of gemcitabine or cisplatin in lung cancer cells (Lovejoy et al., 2012).
Bufexamac and Tubastatin A	HDAC10 inhibitors and inhibitors of lysosomal exocytosis	• Inhibits lysosomal exocytosis independent of Pgp impeding doxorubicin efflux and enhances DNA damage in neuroblastoma (Ridinger et al., 2018). • Disrupts autophagy and promotes doxorubicin’s cytotoxic effect in neuroblastoma cell lines (Oehme et al., 2013).

## Chemoresistance and Cancer Evasion Through Lysosomal Pathways

Most chemotherapeutics used in the clinic are lipophilic, weak-base drugs that can readily be sequestered in the acidic lysosomal compartment. Once in lysosomes, these unprotonated amine-containing compounds are rapidly protonated and remain trapped in these organelles, diminishing their cytotoxic effect (Zhai and El Hiani, 2020). The lysosomotropic nature of several drugs that are widely used in the clinic because of their superior bioavailability and pharmacokinetic parameters poses a major hurdle for cancer treatment. If these drugs do not destabilize lysosomes and cause LMP-mediated cell death, their lysosomal sequestration leads to decreased efficacy and requires higher dosage to reach their cytotoxic concentration. The latter results in increased side effects in patients and chemotherapy resistance.

In the course of transformation, tumor cells may utilize lysosome-centered pathways to evade the effects of chemotherapy. The best characterized class of integral membrane proteins at the PM and the lysosomal membrane, which confer multidrug resistance (MDR: simultaneous insensitivity to different anti-cancer agents) are the P-glycoprotein (Pgp) and other ABC (ATP-binding cassette) transporters (Zhitomirsky and Assaraf, 2016). Tumor cells expressing MDR transporters effectively efflux lysosomotropic ionizable drugs that diffuse into the cytosol or are sequestered in lysosomes. Examples of hydrophobic, weak-base chemotherapeutics that are both Pgp substrates and lysosomotropic are doxorubicin, daunorubicin, vinblastine, sunitinib, vincristine, cisplatin, and sorafenib (Yamagishi et al., 2013; Colombo et al., 2014; Zhitomirsky and Assaraf, 2016; Geisslinger et al., 2020; Zhai and El Hiani, 2020). Treatment of tumor cells with these compounds induces expansion of the lysosomal system, thereby enhancing their lysosomal sequestration and drug resistance (Groth-Pedersen et al., 2007; Zhitomirsky and Assaraf, 2015; Zhao et al., 2020). Mechanistically, this phenomenon was explained by a drug-mediated efflux of lysosomal Ca^2+^ via the Ca^2+^ channel TRPML1 and consequent activation of calcineurin, which by dephosphorylation of TFEB causes its nuclear translocation and activation of lysosomal gene expression (Groth-Pedersen et al., 2007; Zhitomirsky and Assaraf, 2015; Zhitomirsky et al., 2018; Zhao et al., 2020). These and other studies have sparked the interest of cancer biologists on the role of lysosomal Ca^2+^ channels, including TPCs (two-pore channels) and TRPMLs, in cancer progression. However, the molecular mechanisms linking Ca^2+^ dysregulation to tumorigenesis and/or metastatic growth have not been fully elucidated yet. So far, most of the findings appear to be correlative. Increased expression of TPCs has been implicated in cancer cell migration and dissemination in bladder, liver and hematological tumors, while genetic and pharmacologic inhibition of TPCs has been linked to diminished adhesion and migration of invasive tumor cells and formation of lung metastases in a breast cancer mouse model (Nguyen et al., 2017; Faris et al., 2018; Alharbi and Parrington, 2019). Similarly, increased TRPML1 expression in head and neck squamous cell and bladder urothelial carcinoma inversely correlated with patient prognosis, and has been associated with chemotherapy resistance in endometrial adenocarcinoma cells (Faris et al., 2018; Jung et al., 2019; Santoni et al., 2020).

Another cellular mechanism hijacked by tumor cells to evade chemotherapeutics independently of the expression of MDR transporters, is based on their ability to efflux lysosomotropic drugs via upregulation of lysosomal exocytosis (Figure 6). Evidence of such mechanism has been obtained in aggressive rhabdomyosarcoma cells that were shown to effectively purge lysosome-trapped doxorubicin extracellularly via unrestrained lysosomal exocytosis (Machado et al., 2015). This study also identified this pathway as a suitable target for therapeutic intervention, because inhibiting lysosomal exocytosis by verapamil, an FDA-approved Ca^2+^ channel blocker, rendered rhabdomyosarcoma cells sensitive to doxorubicin (Machado et al., 2015). Thus, the use of verapamil in combination with other chemotherapeutics may represent a promising approach to potentiate the cytotoxic effect of some of the lysosomotropic drugs (Colombo et al., 2014; Wong et al., 2015; Table 1). Other strategies have explored the impact of destabilizing cancer cell lysosomes by raising their luminal pH with agents like the anti-malarian chloroquine/hydroxychloroquine/mefloquine or the macrolide antibiotic bafilomycin A, all of which function by inhibiting the lysosomal proton pump v-ATPase. These treatments sensitize metastatic cancer cells to chemotherapeutics and inhibit cancer progression (Circu et al., 2017; Collins and Forgac, 2018; Morgan et al., 2018; Whitton et al., 2018; Table 1).

Lastly, considering that lysosomes control the degradation of cellular constituents and organelles after their fusion with autophagosomes, it follows that disturbance of lysosomal pathways also affects autophagic processes, thereby promoting cancer progression and chemoresistance. In cancer, autophagy has been shown to have a dychotomous function because it can either promote or inhibit tumor growth. This seemingly contrasting role of autophagy is most likely dependent on the tumor type, tumor stage and the pool of oncogenic drivers, as eloquently discussed in numerous recent reviews (Poillet-Perez and White, 2019; Chavez-Dominguez et al., 2020; Mulcahy Levy and Thorburn, 2020; Towers et al., 2020).

In addition to a cell autonomous role in cancer, autophagy induced during cancer therapy coupled to lysosomal degradation has been recognized as a key mechanism of immunosurveillance and resistance to immunotherapy. In a recent report, activation of autophagy has been linked to selective lysosomal degradation of MHC-I and immune-evasion of pancreatic cancer cells. Reduced expression of MHC-I at the cell surface of cancer cells results in failed recognition of these cells by CD8+ T cells, hampering the efficacy of immunotherapy (Yamamoto et al., 2020b). In contrast, inhibition of autophagy and lysosomal degradation restores surface levels of MHC-I, leading to improved antigen presentation and enhanced anti-tumor T cell response. In this model, inhibitors of autophagy sensitize tumors to immune checkpoint blockade therapy (Yamamoto et al., 2020a,b). Thus, inhibition of autophagy and lysosomal degradation may improve immune surveillance and prevent cancer resistance.

## Lysosome Membrane Contact Sites: Future Perspective

It is now widely accepted that in order to communicate and exchange molecules between organelles without engaging in fusion events lysosomes tether at MCS (Prinz et al., 2020). These specialized membrane microdomains are bona fide signaling hubs that allow for the rapid exchange/transfer of lipids, ions and other molecules between the two apposing membranes. Given its extensive membranous network, it is not surprising that the ER forms MCS with virtually every other cellular organelle, including lysosomes/endosomes (Phillips and Voeltz, 2016; Prinz et al., 2020). The lipid and protein compositions of MCS are not only dictated by the characteristics of the individual membranes, but also reflect specific functions that need to take place at these microdomains (Prinz et al., 2020). The most recognized functions of MCS are biosynthesis/transport of lipids (particularly phospholipids and cholesterol) and ions, such as Ca^2+^ (Prinz et al., 2020; Vance, 2020), which are crucial for maintaining the metabolic state of the cell and can be hijacked by cancer cells during malignant transformation.

### Lysosome-ER Membrane Contact Sites

It has been established that more than 99% of late endosomes/lysosomes form dynamic contacts with the ER (Friedman et al., 2013). Although these MCS have been implicated in endosomal tubulation and lipid trafficking, their regulation and formation is still poorly understood. Several studies have described some of the proteins required for the establishment of these MCS, which not only function in cholesterol and lipid trafficking but also in endo-lysosomal positioning. One of these proteins is the ER-localized protrudin, which tethers the ER to the lysosomal membrane by directly binding to Rab7 and phosphatidylinositol 3-phosphate. By subsequent interaction with the Rab7 effector FYCO1 (FYVE-coiled coil-domain-containing protein) and KIF1, protrudin also promotes the movement of endosomes/lysosomes to the cell periphery (Matsuzaki et al., 2011; Raiborg et al., 2016). Interestingly, in a 3D cell culture model of invasive breast cancer cells, overexpression of protrudin has been shown to facilitate late endosome/lysosome translocation to invadopodia. This process regulates invadopodia growth and exocytosis of the metalloprotease MMP14, leading to increased ECM degradation and invasive migration (Pedersen et al., 2020).

Other known constituents of the ER/endo-lysosome MCS are VAPA and VAPB (VAMP associated protein A and B) on the ER side, which interact with ORP1L and STARD3 (steroidogenic acute regulatory protein-related lipid transfer domain protein 3) on the late endosome/lysosome side (Prinz et al., 2020). In primary breast cancer cells, STARD3 overexpression results in increased cholesterol biosynthesis and redistribution of cholesterol at the PM, a phenomenon that correlates with increased Src/FAK signaling and enhanced cancer aggressiveness (Vassilev et al., 2015). Given that STARD3 is a component of the ER/endo-lysosome MCS, it is likely that these contact sites regulate cholesterol mediated signaling during cancer progression.

The cholesterol concentration at the ER/endo-lysosome MCS has recently emerged as an additional regulator of mTORC1 activity. This is mediated by the interaction between VAPs at the ER side with OSBP at the lysosomal side, which facilitates cholesterol transfer from the ER to the lysosomal membrane. Low cholesterol concentration inhibits the interaction of mTORC1 with the Rag GTPases, retaining it in an inactive state in the cytosol. Instead, high cholesterol induces the rapid relocation of mTORC1 to the lysosomal membrane in close proximity to the Rag-GTPase. These proteins sense the cholesterol content of the lysosomal limiting membrane through the amino acid carrier SLC38A9. A negative regulator of this pathway is the Niemann Pick type-C 1 (NPC1) protein, which transports cholesterol from late endosomes/lysosomes to other membrane organelles, including the ER (Platt et al., 2018; Hoglinger et al., 2019; Lim et al., 2019). Mutations in NPC1, causing the neurodegenerative LSD Niemann-Pick type C, result in accumulation of cholesterol at the lysosomal membrane, likely at lysosome MCS, leading to constitutive activation of mTORC1 signaling (Lim et al., 2019).

In addition to lipid transfer, ER/endo-lysosome MCS regulate Ca^2+^ flux between these organelles through engagement of the IP3Rs on the ER side. This activity has direct functional implications for endo-lysosomal fusion and fission and lysosomal positioning (Atakpa et al., 2018). Although not yet proven, we anticipate that cancer cells use these MCS to hyperactivate mTORC1 and decouple transfer of Ca^2+^ between the ER and the endo-lysosomal system, thereby evading Ca^2+^-mediated activation of cell death.

### Lysosome-Mitochondria Membrane Contact Sites

MCS between the lysosomes and the mitochondria have only recently been identified morphologically and function in mitochondrial dynamics and transfer of Ca^2+^ between the organelles (Wong et al., 2019; Peng et al., 2020). Although most of the constituents that establish the tethering of these MCS in mammalian cells are currently unknown, the only recognized protein at this MCS is Rab7 in its GTP-bound state. Inactivation of Rab7 by hydrolysis of GTP leads to disassembly of these contact sites (Wong et al., 2019). Considering the role of Rab7 in lysosomal positioning, we hypothesize that during cancer progression reduced levels of Rab7 promote anterograde movement of lysosomes to the PM prior to lysosomal exocytosis. Uncoupling of the two organelles in cancer cells will also reduce the Ca^2+^ flux from the lysosome to the mitochondria, allowing them to evade mitochondria-mediated apoptosis.

## Conclusion

In response to intrinsic and extrinsic cues, cancer cells undergo transformation, acquire plasticity and become invasive and migratory, features that enable them to escape their primary niche, travel to distant sites and initiate metastatic growth. Although these progressive malignant traits have been known for decades, the factors that regulate their initiation have not been fully elucidated. It is increasingly apparent, however, that during transformation tumor cells reprogram and exploit the lysosomal system to their advantage. By effectively hijacking key lysosome-controlled pathways, cancer cells coordinate energy production, cell survival, immune evasion, proliferation, invasion, metastasis and drug resistance. Thus, dissecting the multiple roles of the lysosomal system in cancer progression may offer additional and out-of-the-box means to treat aggressive and intractable cancers with novel or repurposed therapies. Although we only uncovered the tip of the iceberg and much remains to be discovered, we predict that a lysosome-centric approach to cancer biology will lead to a better understanding of the course of aggressive cancer and pave the way for the development of novel therapeutic drugs.

## Author Contributions

All authors listed have made a substantial, direct and intellectual contribution to the work, and approved it for publication.

## Conflict of Interest

The authors declare that the research was conducted in the absence of any commercial or financial relationships that could be construed as a potential conflict of interest.
